# Congenital diarrhea and enteropathies caused by a heterozygous mutation in the *GUCY2C* gene: a rare case report

**DOI:** 10.3389/fped.2025.1649272

**Published:** 2026-01-13

**Authors:** Chuanjie Yuan, Juanjuan Lyu, Xiaomei Sun, Jin Wu, Ying Liu

**Affiliations:** 1 Department of Pediatrics, West China Second University Hospital, Sichuan University, Chengdu, China; 2 Key Laboratory of Birth Defects and Related Diseases of Women and Children (Sichuan University), Ministry of Education, Chengdu, China

**Keywords:** congenital diarrhea and enteropathies (CODEs), *GUCY2C*, heterozygous mutation, infant, secretory diarrhea

## Abstract

**Objectives:**

This report presents a rare case of congenital diarrhea and enteropathies in a Chinese infant caused by a heterozygous mutation in the *GUCY2C* gene.

**Case presentation:**

A male infant was born prematurely with a history of polyhydramnios and prenatal intestinal dilation. He developed persistent abdominal distension, secretory diarrhea, metabolic acidosis, severe electrolyte imbalances, and failure to thrive. Whole-exome sequencing identified a heterozygous mutation in *GUCY2C* (c.2356T > C, p.Y786H), classified as likely pathogenic. The patient also exhibited intestinal malrotation but did not require surgical intervention. Management included total parenteral nutrition, transition to an amino acid-based formula, and extensive electrolyte supplementation. At the 72-month follow-up, he exhibited normal growth and complete resolution of gastrointestinal symptoms.

**Conclusions:**

A heterozygous mutation in the *GUCY2C* gene (c.2356T > C) was identified as the cause of this rare congenital diarrheal disorder. These findings emphasize the essential function of genetic testing in children with chronic, treatment-refractory diarrhea and highlight the potential for excellent long-term outcomes with suitable supportive care.

## Introduction

Diarrhea in neonates is most commonly secondary to infection or food protein allergy, typically resolving without complications following appropriate treatment. However, a small proportion of infants suffer from congenital enteropathies resulting from monogenic disorders, collectively termed congenital diarrhea and enteropathies (CODEs) (1, 2). The clinical manifestations of CODEs include severe and often life-threatening diarrhea in neonates and infants, frequently accompanied by intestinal dilation and other gastrointestinal abnormalities. Management is challenging and usually requires aggressive fluid resuscitation and total parenteral nutrition (TPN). To date, over 48 different genes have been associated with CODEs, which can be classified into five categories based on the underlying molecular defect: Epithelial nutrient/electrolyte transport, enzymes/metabolism, trafficking/polarity, endocrine cell dysfunction, and immune dysregulation–associated enteropathy (3, 4).

The *guanylate cyclase 2C* (*GUCY2C*) gene encodes the transmembrane receptor guanylate cyclase C (GC-C), a key regulator of intestinal fluid and electrolyte homeostasis. Autosomal dominant CODEs linked to *GUCY2C* mutations are characterized by early-onset chronic secretory diarrhea, abdominal distension, dehydration, metabolic acidosis, and electrolyte disturbances. Other possible complications include intestinal obstruction, volvulus, inflammatory bowel disease (IBD), and urolithiasis (5, 6). Currently, treatment is primarily symptomatic, focusing on maintaining fluid, electrolyte, and acid-base balance. With careful management, long-term outcomes are generally favorable (5, 6). This study presents a rare case of a Chinese infant diagnosed with CODEs attributed to a heterozygous mutation in the *GUCY2C* gene, hence contributing to the genotypic and phenotypic diversity of this disorder.

## Case presentation

A male infant was the first child of non-consanguineous parents. The pregnancy was complicated by polyhydramnios. A fetal ultrasound at 30 + 4 weeks of gestation revealed extensive dilation of the mid to lower abdominal bowel, suggesting the possibility of intestinal atresia. The infant was delivered via cesarean section at 34 + 3 weeks, due to premature rupture of membranes and subsequent fetal distress. The birth weight was 2,420 g, with Apgar scores of 9, 10, and 10 at 1, 5, and 10 min, respectively. Approximately 5,000 mL of pale-yellow amniotic fluid was observed.

**Figure 1:**
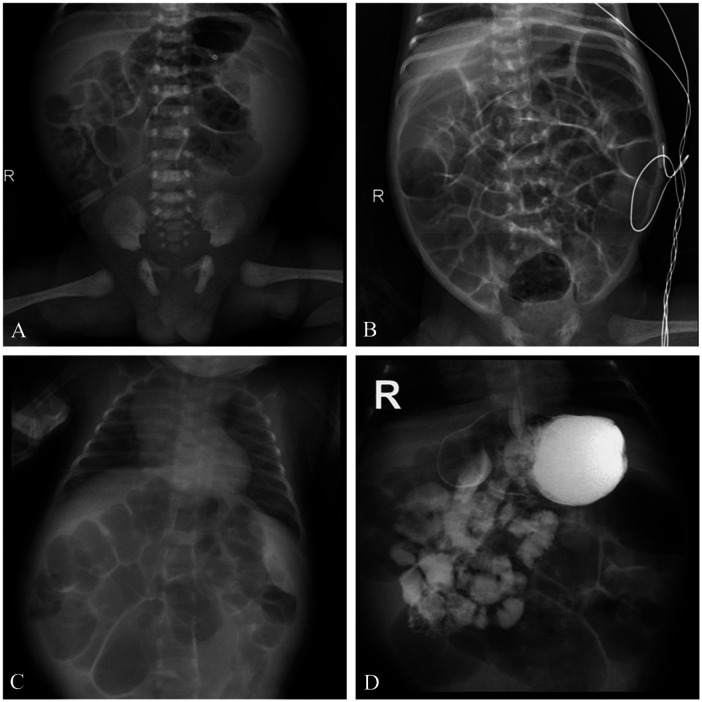
Abdominal x-ray; **(A)** Day 1 of life, **(B)** Day 7 of life, **(C)** Day 14 of life, **(D)** oral small bowel radiography. The abdominal x-ray on days 1, 7, and 14 of life revealed intestinal. Oral small bowel radiography at 70 days of life revealed a reversal of the jejunal and ileal locations, with right-sided jejunum and left-sided ileum.

The patient was promptly admitted to the neonatal intensive care unit. He was kept nothing by mouth, and an extensive diagnostic evaluation was commenced. An abdominal x-ray indicated dilated intestinal loops (Figure 1A), while an abdominal ultrasound was unremarkable. Following the enema, stool passage was unobstructed, and the pediatric surgery team determined that surgical intervention was unnecessary. The infant's weight decreased rapidly to 1.8 kg by the second day of life. Physical examination revealed abdominal distension with visible intestinal patterns and active bowel sounds. Persistent abdominal distension was confirmed on radiographs at 7 and 14 days of life (Figures 1B,C). The infant subsequently developed severe watery diarrhea, resulting in significant metabolic acidosis, electrolyte derangements, renal impairment, and severe anemia.

Laboratory investigations revealed a hemoglobin level of 77 g/L. Urinalysis indicated a pH of 6.5, accompanied by proteinuria (1+), glycosuria (3+), and an elevated urine protein-to-creatinine ratio of 2.72 (reference <0.2). Stool examinations consistently indicated negative results for leukocytes, red blood cells, and pus cells, while testing positive for occult blood. The stool pH measured 7.5, and stool osmolality was below 50 mOsm/kg, indicating secretory diarrhea. The stool culture indicated no bacterial growth. The fasting blood glucose level was 4.0 mmol/L. Arterial blood gas analysis revealed severe metabolic acidosis with a nadir pH of 7.09 (reference range: 7.35–7.45), bicarbonate $\left(\mathrm{HC}O_{3}^{-}\right)$ of 7 mmol/L (reference range: 18–24 mmol/L), and base excess (BE) of −21.2 mmol/L (reference range: −3 to +3 mmol/L). Severe electrolyte disturbances included potassium of 2.3 mmol/L (reference range: 3.5–5.5 mmol/L), sodium at 118 mmol/L (reference range: 135–145 mmol/L), and chloride at 69.4 mmol/L (reference range: 99–110 mmol/L). Serum creatinine and urea levels were elevated at 204 µmol/L (reference range: 17.3–54.6 µmol/L) and 28.77 mmol/L (reference range: 3.2–7.1 mmol/L), respectively.

The initial diagnoses of gastrointestinal anomaly and renal tubular acidosis did not fully explain the persistent and severe clinical picture. A multidisciplinary team review suggested CODEs as a potential etiology. Since the parents declined an intestinal biopsy, whole-exome sequencing (WES) was initiated on day 31 of life, with results available one month later.

The patient received total TPN for 7 days, thereafter transitioning to partial enteral nutrition (PEN) and undergoing a trial of oral feeding using an amino acid-based formula. Enteral feeds were progressively increased as PEN was weaned, guided by feeding tolerance. By day 45, the infant had completely transitioned to an amino acid formula supplemented with breast milk, attaining an intake of 200 mL/kg/day. He received oral potassium citrate and sodium citrate, alongside intravenous sodium bicarbonate, potassium chloride, and sodium chloride. A single transfusion of packed red blood cells was administered during hospitalization. After 51 days, the patient experienced a weight gain of 2.4 kg, and he was subsequently discharged on long-term oral supplements, including potassium citrate, sodium bicarbonate, probiotics, iron, and vitamin D.

**Figure 2:**
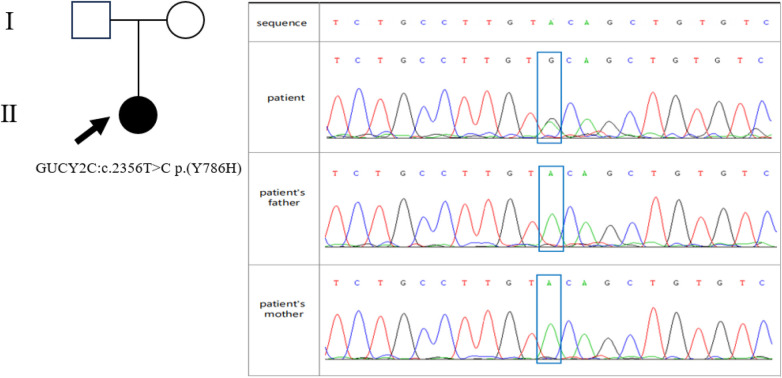
Pedigrees of the identified variant (c.2356T > C). The proband is indicated by black arrows. The Sanger sequencing results for the *GUCY2C* gene in the patient and his parents.

At 66 days of life, the infant was readmitted following a two-day history of vomiting and fever, with a peak temperature of 38.8 °C. He passed yellow, mushy stools 3–5 times daily without blood or mucus. Upon assessment, he weighed 3.1 kg and measured 53 cm in length (BMI 11.04 kg/m^2^, consistent with severe malnutrition). He appeared lethargic, with reduced subcutaneous fat. The abdomen was soft but distended, with palpable bowel loops. Laboratory tests indicated hypokalemia (2.51 mmol/L), elevated inflammatory markers [C-reactive protein(CRP) > 150 mg/L, procalcitonin(PCT) 11.2 ng/mL], anemia (Hb 64 g/L), and persistent metabolic acidosis (pH 7.28, $\mathrm{HC}O_{3}^{-}$ 13.3 mmol/L, BE −14.8 mmol/L). Blood, stool, and urine cultures were negative. On day 70, a small bowel follow-through study revealed intestinal malrotation (Figure 1D). WES results identified a heterozygous mutation in the *GUCY2C* gene: c.2356T > C in exon 21, resulting in a p.Tyr786His (Y786H) substitution. Sanger sequencing confirmed this variant was absent in both parents (Figure 2). According to the 2015 American College of Medical Genetics and Genomics Standards, this variant was classified as “likely pathogenic”. The final diagnoses were *GUCY2C*-CODEs, intestinal malrotation, sepsis, malnutrition, electrolyte imbalance, and anemia. Management during this admission included infection control with ceftriaxone, discontinuation of potassium citrate, oral supplementation with sodium bicarbonate and potassium chloride, another packed red blood cell transfusion, and supportive care with probiotics and smectite.

**Figure 3:**
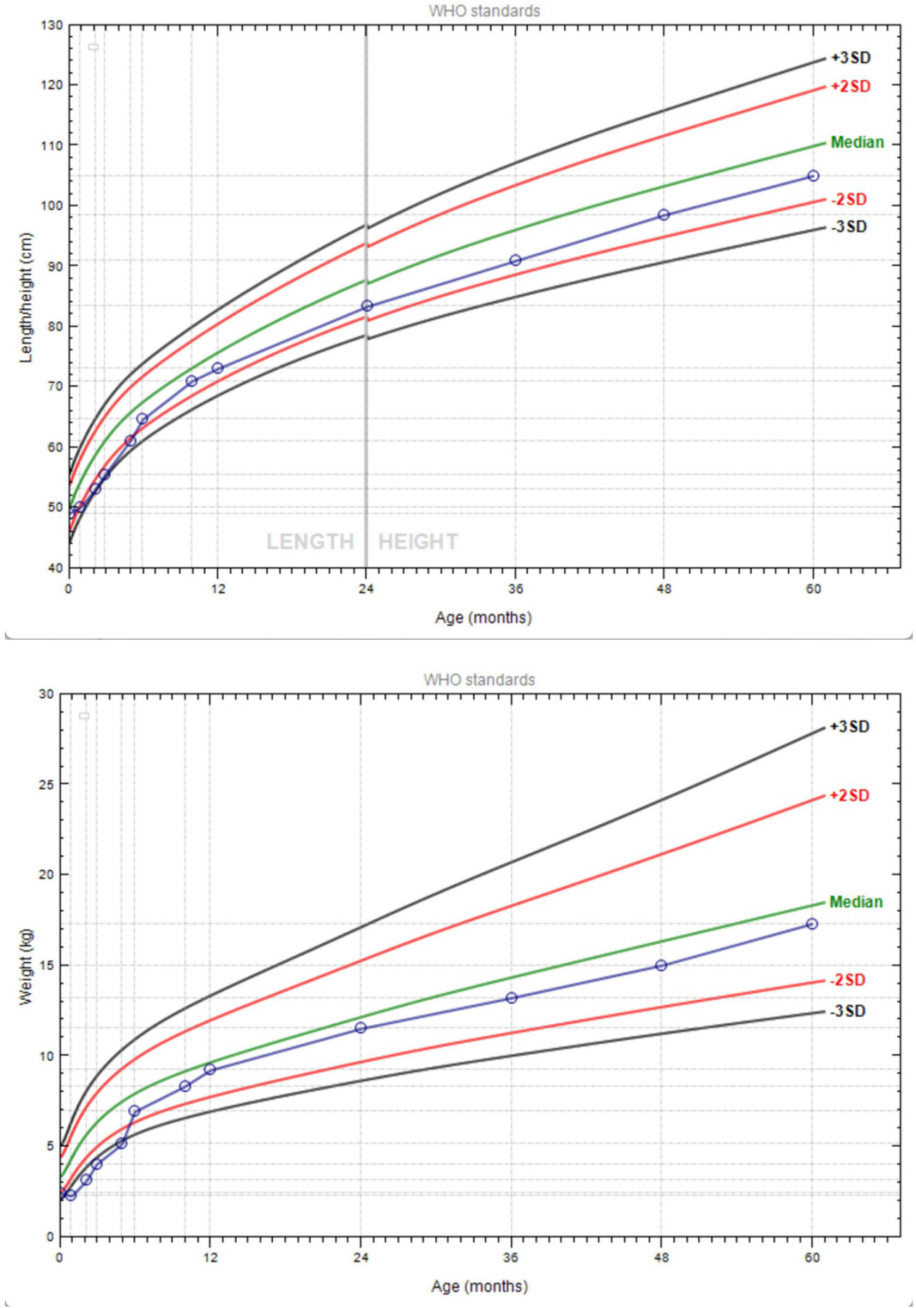
Growth chart of the patient.

At the 4-month follow-up, the patient experienced monthly diarrhea episodes lasting 3–5 days, managed symptomatically at home. The patient exhibited catch-up growth at one year of age, with a height of 73 cm and a weight of 9.2 kg (Figure 3). His diet was diversified to a standard infant formula. At the most recent follow-up (72 months), the patient was thriving, measuring 112.5 cm and 20 kg, with no abdominal distension, pain, or diarrhea. He had 1–2 normal-consistency bowel movements per day. Laboratory tests revealed no anemia, and inflammatory markers [CRP, erythrocyte sedimentation rate (ESR)] were normal, with no evidence of IBD.

## Discussion

The *GUCY2C* gene, located on chromosome 12p12.3, encodes the transmembrane receptor GC-C, mostly expressed in the intestinal epithelium (5). GC-C activation by endogenous ligands (guanylin and uroguanylin) or bacterial enterotoxins triggers intracellular cyclic guanosine monophosphate (cGMP) production. The cGMP activates cGMP-dependent protein kinase II (PKGII) and inhibits the cyclic adenosine monophosphate (cAMP) phosphodiesterase PDE3, resulting in indirect activation of cAMP-dependent protein kinase A (PKA). PKGII and PKA phosphorylate the cystic fibrosis transmembrane conductance regulator (CFTR), resulting in increased chloride secretion. Concurrently, elevated cGMP levels inhibit electroneutral sodium Na^+^/H^+^ exchanger 3(NHE3), reducing sodium absorption and, together with elevated chloride secretion, leading to fluid loss and diarrhea (3, 7, 8). Meanwhile, transmembrane GC-C is a key mediator of acid-stimulated duodenal $\mathrm{HC}O_{3}^{\text{–}}$ secretion (9). Those pathophysiologies underlie the secretory diarrhea and electrolyte depletion seen in *GUCY2C*-CODEs. In this case, genetic analysis via WES was pivotal for diagnosis, particularly given the parents' reluctance to undergo an intestinal biopsy. WES non-invasively identified the *GUCY2C* (c.2356T > C, p.Y786H) mutation, highlighting its utility as a first- or second-tier diagnostic tool for severe, unexplained infantile diarrhea.

The GC-C protein structure comprises an extracellular ligand-binding domain, a transmembrane domain, a kinase homology domain, and a linker region (5). Several mutations in this linker region (p.L775P, p.R792S, p.Asp794Val) have been confirmed as gain-of-function, enhancing cGMP production (5, 6, 10). Although no functional data are available for the p.Y786H variant, its location within the same linker region suggests that it may also confer a gain-of-function. This hypothesis requires formal validation through functional studies.

To date, 10 different autosomal dominant *GUCY2C* mutations have been reported in 57 cases (Table 1) (5, 6, 10–16). The phenotype consistently includes neonatal secretory diarrhea, abdominal distension, electrolyte imbalances, acidosis, and often intestinal dilation, sometimes requiring surgery. This case did not experience intestinal obstruction or acute abdomen and never required exploratory laparotomy. This case presents unique features of intestinal malrotation, a structural anomaly not frequently highlighted in previous reports. Additionally, transient renal impairment and significant proteinuria during the initial acute phase were notable in this case. This could be attributed to pre-renal acute kidney injury from severe dehydration and electrolyte shifts, a known complication of profound secretory diarrhea.

**Table 1:** Summary of currently known autosomal dominant heterozygous mutation in *GUCY2C*.

Reference	Patient numbers	Nucleotide change	Protein alteration	Protein domain	Ethnicity	Consanguinity	Polyhydramnios	Gender	Birth weight (g)	Histology	Diarrhea	Abdominal distension	Metabolic acidosis	Electrolyte disturbances	Ileus	IBD	Bowel dilatation	Others	Abdominal surgery
Fiskerstrand et al. (5)	32	c.2519G > T	p.Ser840Ile	Catalytic	Norwegian	ND	ND	14/32 females 18/32 males	ND	11/32 normal; 14/32 ND; 1/32 Granulation polyp; 1/32 Eosinophilic cells; 1/32 Transmural ileal inflammation, focal active colitis; 1/32 Transmural ileal inflammation, no granulomas; 1/32 Active colitis all segments, mild ileitis; 1/32 Ileal inflammation Nogranulomas; 1/32 Sigmoidal inflammation	30/32	28/32	ND	15/32	10/32	7/32	17/32		8/32 laparotomy; 5/32 intestinal resection
Müller et al. (6)	1	c.1519A > G	p.K507E	Kinase homology	French/Algerian	No	No	Female	3,280	Schaemic ulcers	Yes	Yes	Yes	Yes	No	No	ND	Spsis	No
	1	c.2324T > C	p.Leu775Pro	Linker region	Dutch	No	Yes	Male	3,310	UC gastroduodenitis	Yes	Yes	Yes	Yes	Yes	Yes	ND	Arthritis	Partial resection of intestine
	1	c.2376G > C	p.Arg792Ser	Linker region	German	No	Yes	Female	2,980	Non-specific focal inflammation	Yes	Yes	Yes	Yes	Yes	No	ND	Spsis	No
	1	c.2548A > G	p.N850D	Catalytic	German/Polish	No	Yes	Female	2,620	Normal	Yes	Yes	Yes	Yes	No	No	ND	Spsis	No
Cugley et al. (12)	1	ND	ND	ND	ND	ND	Yes	Female	1,070	ND	Yes	Yes	ND	Yes	ND	ND	ND	Drusen-like deposits	ND
van Vugt et al. (13)	1	c.2485C > T	p.Thr783Ile	Linker region	Swiss	No	Yes	Male	2,000	ND	Yes	Yes	Yes	Yes	ND	ND	Yes		Double-barrel ileostomy
Wolfe et al. (10)	15	c.2381A > T	p.Asp794Val	Linker region	Mennonite	ND	ND	6/15 females 9/15 males	ND	Chronic duodenitis with ileal and pancolonic inflammation (prohand); 14/15 ND	15/15	ND	ND	ND	1/15	ND	2/15	1/15 spsis	1/15 a bowel resection above ileocecal valve; an intestinal obstruction surgery. a tapering enteroplasty surgery; 1/15 two bowel tapering surgeries; 1/15 rectal prolapse surgery;
Bajracharya et al. (14)	1	c.2324T > G	p.Leu775Arg	Linker region	Asian	ND	Yes	Male	ND	No	Yes	Yes	Yes	Yes	Yes	ND	Yes		Exploratory laparotomy 3 times
Qin et al. (15)	1	c.2536G > A	ND	ND	China	ND	ND	Female	1,930	No	Yes	Yes	Yes	Yes	ND	ND	ND		ND
Thorvilson et al. (16)	1	ND	ND	ND	ND	ND	Yes	ND	2,680	Normal	Yes	Yes	Yes	Yes	ND	ND	Yes		No
Scott et al. (11)	1	c.2356T > C	p.Tyr786His	Linker region	ND	ND	Yes	Male	ND	No	Yes	Yes	Yes	Yes	Yes	ND	Yes		Exploratory laparotomy and small intestinal duplication cyst was resected, creation of an ileostomy.
This case	1	c.2356T > C	p.Tyr786His	Linker region	China	No	Yes	Male	2,420	No	Yes	Yes	Yes	Yes	No	No	Yes	Spsis; intestinal malrotation	No

IBD, Inflammatory bowel disease; ND, No data.

Management of neonatal *GUCY2C*-CODEs relies on TPN, aggressive fluid/electrolyte replacement, and surgical intervention for obstruction when indicated. In our patient, TPN provided essential gut rest and nutrition. The successful transition to an amino acid-based formula is a common strategy to minimize antigenic load. Targeted therapies for *GUCY2C*-CODEs are under investigation. GC-C inhibitors such as BPIPP and SSP2518 have revealed promise in reducing pathological cGMP levels and CFTR-mediated chloride secretion in cellular and organoid models harboring hyperactive GC-C mutations (13, 17, 18). However, clinical efficacy remains to be proven, as an attempt to use the CFTR inhibitory modulator Crofelemer in a similar case did not yield clinical benefit (11).

Notably, our patient achieved an excellent long-term outcome, with complete symptom resolution by 72 months. This supports observations that some CODEs may improve with age, possibly due to intestinal maturation, compensatory mechanisms, or dietary modifications (3). This underscores that, despite a severe and life-threatening neonatal presentation, patients with this specific form of CODEs can attain normal growth and development with dedicated supportive care. Continued long-term monitoring for potential complications such as IBD or persistent bowel dilation is warranted.

## Conclusion

This report describes a rare case of CODEs caused by a heterozygous *GUCY2C* (c.2356T > C, p.Y786H) mutation, expanding the known genotypic and phenotypic spectrum of this disorder. Our findings emphasize the importance of early genetic testing and stool electrolyte analysis in infants presenting with chronic secretory diarrhea, severe metabolic acidosis, and electrolyte disturbances. Prompt diagnosis enables the initiation of appropriate supportive management, which can lead to excellent long-term outcomes, as demonstrated in this patient. Further functional studies and clinical development of targeted GC-C inhibitors are needed to advance the management of this rare condition.

## Data Availability

The datasets presented in this study can be found in online repositories. The names of the repository/repositories and accession number(s) can be found in the article/Supplementary Material.
